# Interdental Bone Level around Immediately Placed Implants at Maxillary and Mandibular Molar Sites: A Retrospective Radiographic Analysis

**DOI:** 10.3390/medicina59101701

**Published:** 2023-09-23

**Authors:** Mohammed Alasqah, Khalid S. Alzahrani, Khalid Gufran

**Affiliations:** 1 Department of Preventive Dental Sciences, College of Dentistry, Prince Sattam Bin Abdulaziz University, Alkharj 11942, Saudi Arabia; k.syed@psau.edu.sa; 2 Department of Preventive Dental Sciences, College of Dentistry, Riyadh Elm University, Riyadh 12734, Saudi Arabia; khaledkinani40@gmail.com

**Keywords:** bone loss, early implant, immediate implant, molar teeth

## Abstract

*Background and Objectives*: The current study aimed to compare the amount of interdental bone loss between early and immediate implant placements. *Materials and Methods*: A total of 16 immediate implants and 16 early implants radiographs were included in the current research. The bone level was assessed at two different stages: at the extraction appointment (T1) and after three to six months of implant placement (T2). A line was drawn from the cemento-enamel junction connecting adjacent teeth to the interdental line connecting the interdental alveolar crest at both stages. The difference between measurements in the T1 and T2 stages is the bone loss measurement for the early implant group. For the immediate implant placement sites, the measurements were taken from the interdental bone crest to the implant platform level. *Results*: A Mann–Whitney U test was performed to evaluate the differences between both groups. The descriptive statistics of the T1 and T2 stages for both groups suggest that the bone loss in the T2 stage was generally higher than T1 stage. The immediate implant group showed higher bone loss compared to the early implant group. Moreover, there was significantly higher bone loss (*p* = 0.039) in the immediate implant group compared to the early implant group. *Conclusions*: The results of this study indicate that immediate implant might have disadvantages over early implant in terms of bone loss after the extraction of maxillary and mandibular molar teeth.

## 1. Introduction

In order to achieve the long-term success in implants, osseointegration plays an important role. After achieving the osseointegration along with the occlusal forces applied to the implant, remodelling of the bone usually occurs. In the first year of implant placement, marginal bone level changes occur due to the functional load applied to the implant. Implant failure or marginal bone loss could occur due to clinical handling, implant hardware, and patients’ cooperation [1]. If the peri-implant bone loss is observed as less than 2 mm in the first year of implant placement and 0.2 mm in the following years, this is considered a successful implant treatment [2].

After the extraction of the tooth, substantial bone loss ensues in the alveolar region. This bone loss not only appeared in the bucco-lingual/palatal dimension but also decreased the buccal bone crest [3,4]. During the first three months of tooth extraction, a total of 30% of buccal bony changes occur, and 50% bone reduction is observed at the 12-month follow-up [3]. Moreover, it has been reported that buccal alveolar bone could be decreased by around 2.2 mm after tooth extraction if no immediate implant is placed on the extracted socket [3].

Immediate implant after tooth extraction contains few advantages including reduced number of surgical procedures, prevention of bone resorption, and decrease in comprehensive treatment time. The immediate implant exhibited a higher success rate in both anterior and posterior teeth. Tooth extraction followed by immediate implant is frequently related to residual bone defect between the residual bone wall and neck of the implant [5].

An alveolar ridge alteration occurs in both horizontal and vertical directions during the healing process of the extracted socket [6]. Placement of an immediate implant in the freshly extracted socket prevents these vertical and horizontal bone losses [7]. The validation of reducing bone resorption after the immediate implant is due to the close adaptation of the socket and dental implant. Even though immediate implant is popular due to the preservation of bone loss [8], it also showed that immediate implant is unable to alter bones without any grafting materials [4]. Moreover, immediate implant could be beneficial for full-arch rehabilitation in long-term studies and considered as a valid option when applied in immediate loading full-arch rehabilitation [9,10].

Immediate implants on molar teeth have become popular for restoring missing teeth and have exhibited a higher clinical success rate [11]. Different factors such as advanced prosthetics, a surgical technique [12], and cone-bean computed tomography (CBCT) [13] aided this success rate. The prime advantage of placing an immediate implant in the molar region is less patient morbidity and less waiting period after tooth extraction. Moreover, it also improves the aesthetic outcome and maintains the bone volume [14].

The perfect time for the placement of immediate implant after the tooth extraction is still debatable due to the osseointegration and primary stability. It revealed that the survival rate of immediate implant in the molar region highly depends on providing atraumatic extraction and maintaining the gap between the implant surface and bone [15]. Meanwhile, the delayed implant placement might reduce implant failure since it allows enough time for alveolar bone remodelling [16]. The stability of the implants in the molar region primarily depends on the area of placing the implant and the quality and quantity of the available bone. The immediate implant placed in the anterior or premolar region showed higher stability due to the lesser load taken by the implants in the smaller socket [17]. Therefore, in the molar region, the stability of the implant is less due to the larger socket size of molar teeth compared to the anterior or premolar teeth [15]. Moreover, a systematic review and meta-analysis showed that delayed placement of implants and immediate implant have similar failure rates [18]. Differences were observed in the reduction in alveolar walls following molar extraction with an implant placed after a few weeks compared to a Group II placement in molars [19]. Therefore, the current study aimed to compare the amount of interdental bone loss between early and immediate implant placement. The null hypothesis of this study was that the II and EI groups have no effects on interdental bone levels.

## 2. Materials and Methods

The current retrospective study was conducted at the College of Dentistry, Riyadh Elm University. The institutional review board of Riyadh Elm University approved this study protocol (FPGRP/2022/708/857/811). Moreover, the study was conducted according to the guidelines of the Declaration of Helsinki.

This was a retrospective radiograph-based study. All the radiographs were attained from the archive of the College of Dentistry, Riyadh Elm University. Periapical radiographs were collected from the two stages of the treatment.

For the immediate implant group, the implant (Noble BioCare, Gutenberg, Sweden) was placed after the extraction, and for the early implant group implant (Noble BioCare, Gutenberg, Sweden) it was placed after 6–8 weeks of extraction. The early implant group was considered as a control group. Radiographs for both groups were retrieved at two different stages. First stage at the extraction appointment (T1) and second stage 3–6 months after implant placement.

Following inclusion criteria, included in this study were age an group of 18–65 years, availability of radiographs with the placement of immediate implant with bone graft and collagen membrane in the maxillary and mandibular molar region, sufficient residual alveolar bone in order to provide the stability of the primary implant, replaced the extracted molar with the implant for dental reason (not periodontal reason), presence of neighbouring teeth to refer interdental bone height, and radiographs with only one implant. On the other hand, radiographs with more than one implant, extraction due to periodontal reasons, pathological conditions around the implant site, and a history of medical conditions such as osteoporosis, diabetes mellitus, chemotherapy, or radiotherapy were excluded from the current study.

Periapical radiographs of 16 cases of immediate implant and 16 cases of early implant were selected for the current study based on the convenience sampling technique. Radiographs were collected from the archive, which were taken between 2017 and 2022. As this is a retrospective study, all the implants were not placed with the same surgeon. The 16 immediate implants cases included seven maxillary and nine mandibular implants. Moreover, 3 maxillary and 13 mandibular implants were selected for the 16 early implants control groups.

**Figure 1:**
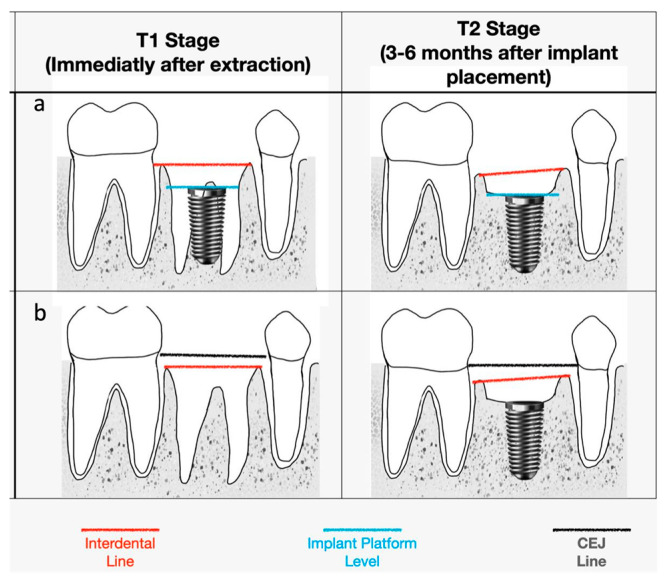
Implant placement in T1 and T2 stage: (**a**) immediate implant and (**b**) early implant.

For the early implant placement site, a line was drawn from the cemento-enamel junction (CEJ) connecting adjacent teeth, and this line is our reference. Another line is the interdental line (IL), a line connecting the interdental alveolar crest. The distance between the two lines was measured at the T1 stage. The same measurement was performed at the T2 stage in the immediate implant group (Figure 1).

At the immediate implant placement site, the distance from IL connects the interdental alveolar crest to implant platform level. The same measurement was performed at the T2 stage in the immediate implant group. In this group, the implant platform level was the reference. Bone loss was compared between the immediate implant group and the early implant group. The differences in bone level measurements were compared and analysed.

Implant diameter for both groups was 4.3. Implant lengths for immediate implant placement were 11/16 10 mm in length and 5/16 13 mm in length. Implant length for early implant placement was 16/16 10 mm in length. Minimum insertion torque for immediate implant placement was 25 N/Cm, and minimum insertion torque for early implant placement was 30 N/Cm. For the early implant group, implants were placed 1 mm sub crestal; for the immediate implant group, measurements of implant depth from the crest was the core of this study, and each implant measured separately. Two independent examiners measured the bone loss using the radiographs two times for the inter-examiner and intra-examiner reliability.

### Statistical Analysis

Statistical analyses were performed using the IBM SPSS software (Chicago, IL, USA) on version 22. Descriptive statistics were performed for both immediate implant and early implant groups. Intra class correlation (ICC) was performed to assess the inter and intra examiner reliability. Normality tests were conducted using the Shapiro–Wilk test to assess the normal distribution of the data. The Mann–Whitney U test was performed to evaluate the differences of bone loss between both groups. The significant level was set at 5%.

## 3. Results

ICC statistics revealed excellent correlation in inter-examiner (ICC = 0.94) and intra-examiner reliability (ICC = 1). The Shapiro–Wilk test showed that the data were not normally distributed; therefore, non-parametric tests were used. The descriptive statistics of the T1 and T2 stages for both groups are presented in Table 1. These statistics suggest that the measurements for bone loss in the T2 stage were generally higher than those for the T1 stage, with a narrower spread around the mean. Moreover, the immediate implant group showed higher bone loss compared to the early implant group.

In the immediate implant group, the minimum difference observed was 0.14, indicating the smallest discrepancy between the T1 and T2 stage measurements, while the maximum difference reached 1.97, representing the largest discrepancy. On average, the difference between the measurements was approximately 0.97. These statistics suggest that, on average, there was a moderate difference between the T1 and T2 measurements, with relatively low variability.

In the early implant group, the minimum difference recorded was 0.18, while the maximum difference was 1.16. The mean difference was found to be 0.57, representing the average changes between the two stages.

**Figure 2:**
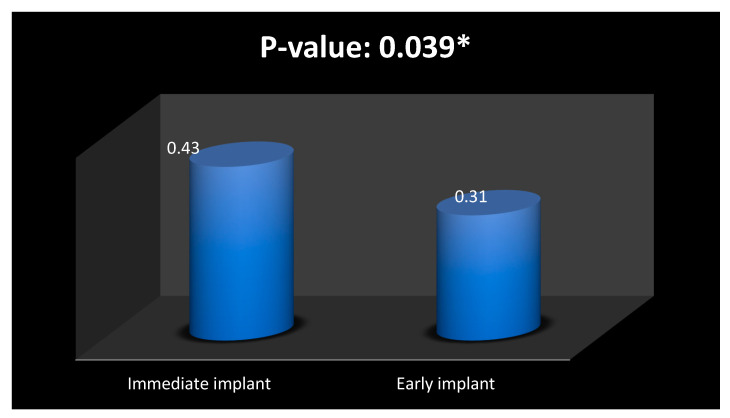
The bone height measured from CEJ. * Significantly different.

The Mann–Whitney U test was performed to compare the measurement of bone loss between the immediate implant and early implant groups. It showed that there was significantly higher bone loss (*p* = 0.039) in the immediate implant group compared to the early implant groups (Figure 2).

## 4. Discussion

In the last 20 years, implant dentistry has advanced significantly, giving practitioners alternatives for dental rehabilitation. Immediate implants are a clinically predictable treatment that prevents post-tooth extraction bone resorption. The current study compared the bone loss for immediate implant and early implant in two different stages; the first stage was placing the implant after the extraction, and the second stage was three to six months after the extraction. The outcome of the current study showed that there was significantly higher bone height in the early implant group compared to the immediate implant group. Therefore, the current study rejects the null hypothesis of the study.

In the current study, the changes in the alveolar width’s horizontal dimension were documented for each patient in both stages. Around any implant, there were no detectable or explorable lingering bone flaws. This finding follows the previous study where Covani et al. [20] found that all immediate implants were asymptomatic, clinically stable, and did not exhibit bone abnormalities during the second stage of surgery.

It was stated in the previous studies that after tooth extraction, two-thirds of the alveolar bone resorbs in the first three months [3,21]. Moreover, it was observed by Cardarpoli et al. [22] that by day 14 of tooth extraction, the majority of the bundle bone is undergoing resorption. Likewise, studies found that more than 20% of buccal plate of the alveolar process was lost during the first 12 weeks of tooth extraction [23,24,25]. These findings may be explained by the loss of the remaining bundle bone following tooth extraction, which makes it non-functional. The current study also exhibited horizontal bone loss in both groups after six months of the extraction.

The current study measured the bone loss on molar teeth from only 16 immediate implants and 16 early implant cases and showed significant differences in bone loss between these two groups. In comparison to this study, Pradhan et al. [26] assessed bone loss in 53 patients in only maxillary central incisors and did not find any significant difference. This contrasting outcome might be due to the differences in the alveolar bone thickness of anterior and posterior teeth.

According to Cosyn et al. [27], there was about 0.12 mm of bone loss after one year and 0.19 mm after five years. Following implant insertion, Raes et al. [28] showed bone gains of 1.01 mm at one year and 0.98 mm at eight years. The current study only observes bone loss after six months. Therefore, the outcome could not be compared with the aforementioned studies. The mean difference after six months was 0.97 and 0.57 for immediate implant and early implant, respectively. Between these three research studies, the figures are different. This could be due to various surgical techniques; for example, no bone transplants were used in the research by Raes et al. [28] and the current study. However, measurements are challenging and might vary across studies since the implant does not fit precisely in the extraction socket, making it difficult to assess bone-to-implant contact at baseline. Remarkably, there were not many changes between the timeline in all these studies, indicating that levels of peri-implant bone stay steady following healing and maturation. A platform-switching implant design with an implant abutment conical connector was employed in the previous investigations [27,28]. It showed that control of micromotion between the implant and abutment is cited as a key issue affecting the stability of crystal bone levels [29].

In the present study, in the experimental group where an immediate implant was placed, the measured value was 0.43. On the other hand, in the control group where an early implant was implemented, the measured value was 0.31. The lower measured value in the control group suggests that an early implant in the molar socket might have a less interdental bone loss compared to an immediate implant placement in molar sites.

The Mann–Whitney U test was used to compare two independent groups. Both an immediate implant and early implant placement were associated with modest peri-implant bone loss in the previous research conducted by Slagter et al. [30] in contrast to the present study. A previous study by Alasabeeha et al. [31] reported two implants (or 5.5%) failed in the group with immediate loading; however, implants placed at six weeks had a 100% success rate. This study did not observe any failure in implant placement in both groups. Another research by El-Sheikh et al. [32] placed two implants in the mandible using a one-stage surgical method and linked the mandibular overdentures to the implants using locator attachments after 10 weeks revealing a similar finding.

For last few years, other commonly adopted procedures in regard to the post-surgical management of the implants have been conducted [9,33,34,35]. Short implant and titled implants are the most common approaches among these. Titled implants have been used to preserve the anatomical structure by using the available native bone [34,36]. While a minimal amount of bone is present, title implants are an appropriate option for full-mouth rehabilitation in an immediate loading of longer implants to enhance the stability [34]. Moreover, a study on extra-short implants reported similar outcomes as longer immediate implants [35].

### Limitations of the Study

The current study included all the radiographs based on the convenient sampling technique. A proper sample size calculation could have strengthened the outcome of the study. Moreover, unlike other studies, the current study did not observe long-term follow-up. This study only assessed bone loss after six months. Long-term follow-up could have provided more insight into the bone loss related to the implant placement in both Group II and early implant cases. In addition, this study did not consider the surgical procedure of the teeth. Surgical extraction causes more bone loss than normal extraction; therefore, surgical procedures should consider this in future studies along with the other limitations of this study. Furthermore, the radiographs taken at T1 and T2 for the immediate implant group might not be at the same angle, and the actual landmark was not reproducible until a radiographic stent was used. This variable could influence the amount of bone loss measurements. Therefore, actual bone loss measurements might slightly vary from the outcome of the study.

## 5. Conclusions

Taken together, these findings suggest that immediate implants might have disadvantages over early implants in terms of achieving higher bone levels. These results contribute to the understanding of the potential of immediate implants in both maxillary and mandibular sites. Further research and clinical studies are warranted to validate and explore these findings in a broader context.

## Figures and Tables

**Table 1:** The mean of bone height at placement and at 6 months after implant placement.

Group	Stages	Minimum (mm)	Maximum (mm)	Mean	SD
II	T1	0.77	5.31	3.01	1.29
	T2	0.24	3.53	2.03	0.96
	T1-T2	0.14	1.97	0.97	0.57
EI	T1	0.88	3.06	1.92	0.68
	T2	1.38	4.22	2.49	0.83
	T1-T2	0.18	1.16	0.57	0.36

mm, millimetre; MD, mean differences; SD, standard deviation; II, immediate implant; EI, early implant; T1, first time placing the implant after the extraction; T2, three to six months after the extraction; and T1-T2, the difference between the two stages.

## Data Availability

Data could be available upon special request to the corresponding author.
